# Hopelessness and Smoking among Black Adults

**DOI:** 10.31586/ojms.2025.1191

**Published:** 2025-02-27

**Authors:** Shervin Assari, Babak Najand, Payam Sheikhattari

**Affiliations:** 1 Department of Internal Medicine, Charles R Drew University of Medicine and Science, Los Angeles, CA, USA; 2 Department of Urban Public Health, Charles R Drew University of Medicine and Science, Los Angeles, CA, USA; 3 Marginalization-related Diminished Returns, Los Angeles, CA, USA; 4 Center for Urban Health Disparities Research and Innovation, Morgan State University, Baltimore, MD, USA; 5 The Prevention Sciences Research Center, School of Community Health and Policy, Morgan State University, Baltimore, MD, USA; 6 Department of Public and Allied Health, School of Community Health and Policy, Morgan State University, Baltimore, MD, USA

**Keywords:** Depression, Hopelessness, Hope, Mental Health, Longitudinal Research, Socioeconomic Disparities, Racial Disparities, Emotional Regulation, Smoking, Tobacco Use

## Abstract

**Background:**

While the link between depression and smoking is known, less is known about the relationship between hopelessness and smoking in large national community-based sample of Black people.

**Aims:**

This study investigates the association between hopelessness and smoking status, using data from the National Survey of American Life (NSAL), which is the only ethnically diverse nationally representative sample of Black adults.

**Methods:**

Data from the NSAL were analyzed. Hopelessness and smoking status were assessed using structured interviews. Logistic regression was employed to assess the link between hopelessness and smoking status, controlling for potential confounders such as demographic factors and socioeconomic indicators as well as depression.

**Results:**

4,939 participants entered our analysis. Hopelessness was significantly associated with higher odds of smoking status. This association remained robust after adjusting for confounders such as demographic factors, socioeconomic status, and depression.

**Conclusions:**

Hopelessness may be a critical risk factor for smoking in Black adults. Promoting hope as a component of targeted tobacco cessation programs may help reduce tobacco use of Black populations.

## 1. Introduction

While depression and tobacco use are linked [1,2], less is known about the role of hope in preventing tobacco use [3]. This is important because if hope is inversely associated with tobacco use, hope provision may be a useful aspect of tobacco cessation programs, families, communities, and schools may use provision of hope as a strategy to tackle tobacco use as a public health pressing concern [4].

Past research has suggested that depression and associated hopelessness may heighten vulnerability to substance use [5–7]. However, the specific effects of these mental health challenges on adolescent tobacco and marijuana use remain insufficiently understood. Moreover, several gaps in the existing literature necessitate further investigation. First, prior studies have often been limited in diversity, with findings primarily derived from White, middle-class samples, raising questions about the generalizability of results to other racial, ethnic, and socioeconomic groups. Additionally, much of the evidence stems from clinical samples or cross-sectional studies, which provide limited insight into the temporal relationship between childhood mental health challenges and adolescent substance use initiation. Finally, the effect of hope on tobacco use of Black Americans remains unclear, particularly when accounting for the interplay between confounding factors.

Gender differences may also play a critical role in the relationship between hope and tobacco use [8]. People may respond and cope to hopelessness and associated depression in distinct ways, influenced by group and gender differences in coping strategies, socialization [9–11]. For example, females are more likely to internalize emotional distress, whereas males are more likely to externalize distress through risk-taking behaviors, including substance use [12–14]. Investigating potential gender differences in the association between smoking and hope is essential for tailoring prevention and intervention efforts to meet the specific needs of both genders.

This study leverages the National Survey of American Life (NSAL) data [15–17] to examine the relationship between hopelessness and ever smoking status among Caribbean Black and African American adults. Specifically, it evaluates whether the hopelessness-smoking link remains significant net of demographic factors, SES indicators, and lifetime depression.

## 2. Methods

### 2.1. Study Design and Sample

This study utilized data from the National Survey of American Life (NSAL) [15–17], a national cross-sectional survey designed to examine the social, economic, and mental health conditions of African Americans, Caribbean Blacks, and non-Hispanic Whites in the United States. The NSAL employed a multistage probability sampling design to ensure adequate representation of key racial and ethnic subgroups. The analytic sample for this study included 4,939 participants who self-identified as African American or Caribbean Black.

For this analysis, we focused on adults aged 18 years and older who had complete data on smoking behaviors, hopelessness, and key demographic and socioeconomic variables. Sampling weights were applied to account for the complex survey design and to ensure national representativeness of the estimates.

### 2.2. Measures

#### 2.2.1. Smoking Behavior

Smoking status was assessed using a binary outcome measure:

**Ever smoked**: Participants self-reported whether they have smoked in their lifetime. Literature has mostly defined people ever smokers if they affirm that they have smoked at least 100 cigarettes in their lifetime.

#### 2.2.2. Hopelessness

To measure hope, we reverse coded a hopelessness measure score. Hopelessness was assessed using a validated scale capturing participants’ feelings of pessimism about the future. Original responses were coded such that higher scores indicated greater levels of hopelessness. Scores ranged from 5 (lowest hopelessness) to 20 (highest hopelessness). For our analysis, we reverse coded this measure to have a measure of hope.

#### 2.2.3. Confounders

Sociodemographic variables were included as covariates to control for potential confounding effects:

**Age** (continuous): Self-reported age in years.
**Gender**: Dichotomized as male or female.
**Ethnicity:** Participants self-identified themselves as African American (no tie to a Caribbean country such as Jamaica) or Caribbean Black (Black people with ancestral ties to Caribbean countries) [18–20].
**Lifetime major depressive episode:** Using CIDI, endorsement for diagnostic criteria for MDD was recorded as a 0 / 1 variable.
**Education**: Measured in years of schooling (continuous).
**Employment status**: Classified as employed or unemployed.
**Marital status**: Dichotomized as married or not married (including single, divorced, or widowed).

### 2.3. Statistical Analysis

Data were analyzed using Stata software (version 17). Logistic regression models were used to examine the association between hopelessness and smoking behaviors (ever smoked) while adjusting for the confounders listed above. To account for the complex survey design, we incorporated the NSAL survey weights, stratification, and clustering variables in all analyses. Adjusted odds ratios (ORs), standard errors (SEs), 95% confidence intervals (CIs), and p-values were reported for all models. **Model 1**: Association between hope and ever smoking, adjusted for age, gender, and ethnicity. **Model 2**: Association between hope and ever smoking, adjusted for the same demographic covariates as well as SES indicators (education, employment, and marital status). **Model 3**: Association between hope and ever smoking, adjusted for the same demographic and SES covariates as well lifetime history of a major depressive episode (MDE in lifetime based on DSM 4.0). We did not present the results of gender difference because there were no major gender difference in the results. All statistical tests were two-tailed, and significance was determined at the 0.05 level. To ensure robust estimates, we used Stata’s svy: logistic command for survey-weighted logistic regression analyses. Results were presented in tables showing ORs, SEs, 95% CIs, and p-values for each model.

## 3. Results

### 3.1. Minimally adjusted model

As shown in Table 1, hope was significantly associated with lower odds of ever smoking (OR = 0.949, SE = 0.011, 95% CI: 0.928–0.972, p < 0.001), suggesting that individuals with greater feelings of hope were protected against smoked in their lifetime. Male participants had significantly higher odds of ever smoking compared to females (OR = 1.849, SE = 0.171, 95% CI: 1.537–2.225, p < 0.001). Age was also positively associated with ever smoking (OR = 1.025, SE = 0.003, 95% CI: 1.019–1.031, p < 0.001), indicating that older individuals were more likely to report lifetime smoking. Ethnicity emerged as a significant factor, with African Americans having significantly higher odds of ever smoking compared to Caribbean Blacks (OR = 2.191, SE = 0.550, 95% CI: 1.324–3.625, p = 0.003).

### 3.2. Partially adjusted model

As shown by Table 2, hope was significantly associated with lower odds of ever smoking (OR = 0.960, SE = 0.012, 95% CI: 0.936–0.986, p = 0.003). This finding suggests that individuals with higher levels of hope were less likely to report having smoked in their lifetime when controlling for demographic and SES indicators. Among SES indicators, the income-to-poverty ratio was not significantly associated with ever smoking (OR = 0.980, SE = 0.018, 95% CI: 0.945–1.016, p = 0.259). Similarly, being married was not a significant predictor of ever smoking (OR = 1.159, SE = 0.115, 95% CI: 0.951–1.414, p = 0.141). However, unemployment was significantly associated with higher odds of ever smoking (OR = 1.423, SE = 0.198, 95% CI: 1.077–1.881, p = 0.014). Male participants had significantly higher odds of ever smoking compared to females (OR = 1.858, SE = 0.176, 95% CI: 1.537–2.245, p < 0.001). Age was positively associated with ever smoking (OR = 1.025, SE = 0.003, 95% CI: 1.020–1.031, p < 0.001), indicating that older individuals were more likely to report lifetime smoking. Educational attainment was marginally significant, with higher years of schooling associated with lower odds of ever smoking (OR = 0.954, SE = 0.022, 95% CI: 0.910–1.000, p = 0.050). Ethnicity remained a significant factor, with African Americans having higher odds of ever smoking compared to Caribbean Blacks (OR = 2.145, SE = 0.539, 95% CI: 1.296–3.550, p = 0.004).

### 3.3. Fully adjusted model

As shown by Table 3, hope was significantly associated with lower odds of ever smoking (OR = 0.969, SE = 0.013, 95% CI: 0.943–0.995, p = 0.020), indicating that individuals reporting higher levels of hopelessness were less likely to have smoked in their lifetime after controlling for other factors. A history of lifetime depressive episodes (DSM-MDE) was strongly associated with increased odds of ever smoking (OR = 1.635, SE = 0.171, 95% CI: 1.326–2.017, p < 0.001), suggesting a significant link between depressive symptoms and smoking behaviors. Among SES variables, the income-to-poverty ratio was not significantly associated with smoking (OR = 0.976, SE = 0.018, 95% CI: 0.941–1.013, p = 0.197), and marital status was also not a significant predictor (OR = 1.182, SE = 0.122, 95% CI: 0.961–1.454, p = 0.112). However, unemployment was significantly linked to higher odds of ever smoking (OR = 1.411, SE = 0.201, 95% CI: 1.060–1.878, p = 0.019). Demographic variables showed a strong influence, with males having significantly higher odds of ever smoking compared to females (OR = 1.911, SE = 0.178, 95% CI: 1.586–2.303, p < 0.001), and older age was associated with increased odds of smoking (OR = 1.026, SE = 0.003, 95% CI: 1.020–1.032, p < 0.001). Educational attainment emerged as a protective factor, with higher years of schooling associated with lower odds of ever smoking (OR = 0.951, SE = 0.022, 95% CI: 0.907–0.996, p = 0.035). Ethnicity also played a significant role, as African Americans were more likely to have ever smoked compared to Caribbean Blacks (OR = 2.157, SE = 0.559, 95% CI: 1.283–3.627, p = 0.004).

## 4. Discussion

### 4.1. Objective

The primary objective of this study was to examine whether higher levels of hope among adults are associated with lower odds of lifetime cigarette smoking, using data from the National Survey of American Life (NSAL) [15–17]. This research focuses on Caribbean Black and African American adults, a population often overlooked in smoking-related studies, to explore the protective role of hope against smoking behavior. While much of the existing literature examines smoking through a clinical or cross-sectional lens, this study fills a critical gap by investigating hope as a psychological resilience factor that reduces smoking risk. The findings provide important insights into how psychological well-being intersects with smoking behavior in marginalized populations.

### 4.2. Findings

The study’s results indicate that higher levels of hope are strongly associated with a reduced likelihood of lifetime cigarette smoking among both Caribbean Black and African American adults. Individuals with higher hope were less likely to have ever smoked, demonstrating the protective role of psychological resilience in smoking prevention. While hopelessness has been well-documented as a risk factor for smoking, these findings suggest that hope operates as a buffer, reducing the likelihood of individuals initiating or continuing smoking behaviors. Those with higher hope may have better coping mechanisms and less reliance on smoking as a maladaptive response to stress or emotional challenges.

Ethnic differences in smoking prevalence were also observed, with African Americans showing higher rates of smoking compared to Caribbean Blacks. Both groups, however, face unique challenges tied to structural inequities, including systemic racism and limited access to resources like quality healthcare, education, and employment. These inequities may increase hopelessness, contributing to higher smoking rates. Conversely, hope appears to mitigate these risks by fostering psychological resilience, helping individuals navigate structural and emotional challenges.

### 4.3. Mechanisms Behind the Protective Role of Hope

Hope embodies a positive outlook and goal-directed thinking, which may serve as a safeguard against smoking and other risky behaviors. Individuals with higher levels of hope are more likely to engage in proactive health behaviors and less likely to resort to smoking as a coping mechanism. While hopelessness is linked to smoking through psychological, neurobiological, and social pathways, hope appears to counteract these influences.

- **Psychologically**, hope enhances motivation and self-efficacy, encouraging individuals to pursue healthier choices and avoid smoking as a maladaptive behavior.
- **Neurobiologically**, hope may support reward-processing systems, reducing the appeal of nicotine as a source of temporary relief from distress.
- **Socially**, individuals with higher hope are more likely to access supportive networks and resources, which can further discourage smoking.

Together, these pathways illustrate how hope acts as a protective factor against smoking by fostering resilience and reducing the likelihood of harmful behaviors.

### 4.4. Policy Implications

These findings emphasize the importance of fostering hope as part of public health strategies to reduce smoking prevalence among African American and Caribbean Black adults. Policies and interventions should focus on enhancing mental health resilience through community-based initiatives, early identification of individuals at risk, and integration of hope-focused strategies in smoking cessation programs. Policymakers and program developers may wish to incorporate hope-building exercises into counseling and stress management programs. For example, resilience workshops and mindfulness training could help individuals develop strategies to cope with adversity without resorting to smoking. They may establish culturally tailored interventions that leverage community strengths to foster hope and resilience. This includes initiatives that build social support networks and address stressors specific to African American and Caribbean Black populations. They may also address systemic inequities that contribute to hopelessness, such as limited access to quality healthcare, stable employment, and affordable housing. Ensuring equitable access to smoking cessation resources and mental health services is critical for long-term behavioral change.

### 4.5. Limitations

While this study provides valuable insights, it has several limitations. The cross-sectional design prevents definitive conclusions about the causal relationship between hope and smoking behavior; longitudinal data are needed to determine whether higher hope levels lead to reduced smoking or if smoking behavior influences hope. Additionally, self-reported data on smoking and hope may be subject to biases, such as underreporting or recall inaccuracies. The sample, though representative, does not fully account for variations within Caribbean Black and African American populations, such as nativity and cultural practices, which may shape smoking behaviors. Another limitation is our measurement of hope, which was derived from a reverse-coded hopelessness scale rather than a direct assessment of hopefulness. We acknowledge that hope and hopelessness may not be perfect opposites, and future research should validate these findings using direct measures of hope and hopefulness. Furthermore, this study focused exclusively on hope as a psychological factor without examining its interaction with other mental health conditions, such as depression or anxiety. Finally, geographic and policy differences that could influence smoking behaviors and access to cessation resources were not explicitly addressed, highlighting the need for further research in diverse contexts.

### 4.6. Future Directions

Future research should use longitudinal designs to establish the causal role of hope in smoking prevention. Studies should also explore how hope interacts with other protective factors, such as social support and cultural resilience, to buffer against smoking. Examining the interplay between hope and structural determinants, such as access to resources and exposure to systemic inequities, would provide a more comprehensive understanding of smoking behaviors. Additionally, tailored interventions that consider geographic and policy contexts would enable targeted strategies to reduce smoking disparities among African American and Caribbean Black populations.

## 5. Conclusion

This study highlights hope as a significant protective factor against lifetime cigarette smoking among African American and Caribbean Black adults. By mitigating the influence of structural inequities and psychological distress, hope fosters resilience and reduces reliance on harmful behaviors like smoking. The findings underscore the need for integrated public health and mental health strategies to address smoking disparities in marginalized populations. Investments in equitable policies, community-based programs, and resilience-building interventions are essential for reducing smoking prevalence and improving health outcomes in these communities.

## Figures and Tables

**Table 1.** Results of Model 1

	OR	SE	95%	CI	p
Hope	0.949	0.011	0.928	0.972	< 0.001
Male	1.849	0.171	1.537	2.225	< 0.001
Age	1.025	0.003	1.019	1.031	< 0.001
Ethnicity					
African American	2.191	0.550	1.324	3.625	0.003
Intercept	0.072	0.020	0.042	0.125	< 0.001

Source: NSAL: Sample: Black People, Outcome: Ever Smoking; Model 3 controlled for demographic factors, SES indicators, and MDE (DSM_MDE)

**Table 2.** Results of Model 2

	OR	SE	95%	CI	P
Hope	0.960	0.012	0.936	0.986	0.003
Income to Poverty Ratio	0.980	0.018	0.945	1.016	0.259
Married	1.159	0.115	0.951	1.414	0.141
Unemployed	1.423	0.198	1.077	1.881	0.014
Male	1.858	0.176	1.537	2.245	< 0.001
AGE	1.025	0.003	1.020	1.031	< 0.001
Educational Attainment (Years of Schooling)	0.954	0.022	0.910	1.000	0.050
Ethnicity					
African American	2.145	0.539	1.296	3.550	0.004
Intercept	0.132	0.053	0.059	0.295	< 0.001

Source: NSAL: Sample: Black People, Outcome: Ever Smoking; Model 3 controlled for demographic factors, SES indicators, and MDE (DSM_MDE)

**Table 3.** Results of Model 3

	Odds ratio	std. err.	[95% conf.	interval]	P>t
Hope	0.969	0.013	0.943	0.995	0.020
Lifetime Depressive Episode (DSM)	1.635	0.171	1.326	2.017	< 0.001
Income to Poverty Ratio	0.976	0.018	0.941	1.013	0.197
Married	1.182	0.122	0.961	1.454	0.112
Unemployed	1.411	0.201	1.060	1.878	0.019
Male	1.911	0.178	1.586	2.303	< 0.001
Age	1.026	0.003	1.020	1.032	< 0.001
Educational Attainment (Years of Schooling)	0.951	0.022	0.907	0.996	0.035
Ethnicity					
African American	2.157	0.559	1.283	3.627	0.004
Intercept	0.128	0.051	0.057	0.286	< 0.001

Source: NSAL: Sample: Black People, Outcome: Ever Smoking; Model 3 controlled for demographic factors, SES indicators, and MDE (DSM_MDE)
